# Inhibition of RNF6 alleviates traumatic brain injury by suppressing STAT3 signaling in rats

**DOI:** 10.1002/brb3.1847

**Published:** 2020-09-21

**Authors:** Bin Liu, Gang Zhang, Shukun Cui, Guoliang Du

**Affiliations:** ^1^ Department of Neurosurgery Six Cangzhou Central Hospital Cangzhou China

**Keywords:** JAK‐STAT3 signaling pathway, ring finger protein 6, SH2‐containing tyrosine phosphatase‐1 (SHP‐1), traumatic brain injury

## Abstract

**Background:**

Traumatic brain injury (TBI) has ranked as one of the leading causes of disability and death in the world. The neuroinflammation mediated by signal transducer and activator of transcription 3 (STAT3) signaling during the progression of TBI leads to long‐term neurodegeneration. Ring finger protein 6 (RNF‐6) is an E3 ubiquitin ligase and can regulate the activity of STAT3 signaling pathway by targeting its inhibitors. However, the mechanism underlying this process in TBI remains poorly understood.

**Methods:**

In this research, cortical impact injury was used to construct the TBI rat model. Western blot assay was performed to evaluate the protein levels of RNF6, Src homology 2 domain‐containing protein tyrosine phosphatase 1 (SHP‐1), and STAT3/pSTAT3. QRT‐PCR assay was performed to assess the RNA levels of RNF6 and other cytokines. The neural function of TBI rats was estimated by modified Neurological Severity Scores test.

**Results:**

The expression of RNF‐6 was up‐regulated in the brain tissues of TBI rats. Down‐regulation of RNF6 alleviated the symptoms and improved the neural recovery postinjury in TBI rats. Inhibition of RNF6 suppressed the cerebral inflammation by up‐regulating the protein level of SHP‐1 and down‐regulating the phosphorylation level of STAT3.

**Conclusion:**

Inhibition of RNF6 alleviated TBI by suppressing the STAT3 signaling in TBI rats.

## Graphical abstract

1. Inhibition the expression of RNF suppressed the cerebral inflammation. 2. Inhibition of RNF6 alleviated traumatic brain injury.

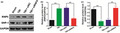

## 1 INTRODUCTION

Traumatic brain injury (TBI) is a devastating actuality in clinic (Oberholzer & Muri, 2019). Approximately 10 million patients in the USA and Europe are estimated to be impaired by permanent physical disability and cognitive impairment caused by TBI (Marshall et al., 2019; Zaninotto et al., 2019). TBI also contributes to neurological diseases including epilepsy, Alzheimer's disease, Parkinson's disease, and chronic neuritis (Gardner et al., 2018). The pathophysiological process of TBI includes the ischemic cascades caused by cerebrovascular injury and blood–brain barrier (BBB) damage caused by neuroinflammation (Ciccia, Beekman, & Ditmars, 2018; Pink, Williams, Alderman, & Stoffels, 2019). Therefore, it is urgent to identify new therapeutic strategies to treat neuroinflammation and alleviate the symptoms of TBI.

The Janus kinase/signal transducer and activator of transcription 3 (JAK/STAT3) signal pathway plays a crucial role in multiple biological activities including cell proliferation, cell differentiation, apoptosis, and inflammation (Raible et al., 2015). Glial cells and neurons produce multiple chemokines in the area of brain trauma, including interleukin 1 (IL‐1), IL‐8, IL‐6, and tumor necrosis factor‐α (TNF‐α) (Zhao, Zhang, Li, Su, & Hang, 2011). These factors activate the JAK/STAT3 signaling pathway to mediate the regional inflammatory reaction thereby worsening the neuroinflammation symptoms (Cui et al., 2017; Gong et al., 2019). Thus, identifying the regulatory mechanism of JAK/STAT3 signaling responsible for the progression of TBI can provide new targets for the treatment of TBI.

The ubiquitin–proteasome system is an important protein quality control mechanism in eukaryotic cells (Xu et al., 2009). Ring finger protein 6 (RNF6) is a zinc finger domain‐containing protein (Xu et al., 2016). As an E3 ubiquitin ligase, RNF6 functions together with E2 ubiquitin binding enzymes to promote the degradation of target proteins (Macdonald et al., 1999). It has been reported that RNF6 was up‐regulated due to the activation of JAK/STAT3 pathway and it could mediate the degradation of SH2‐containing tyrosine phosphatase‐1 (SHP‐1), which is an important STAT3 inhibitor (Huang et al., 2018; Liu et al., 2018). Therefore, the abnormal RNF‐6 regulation may be a cause of abnormal activation of STAT3 pathway and neuroinflammation during TBI.

In this study, we reported that RNF6 was up‐regulated in the cerebral tissues of TBI rats. Inhibition of RNF6 through siRNA alleviated symptoms of TBI including brain edema and neuroinflammation. Mechanically, we demonstrated that inhibition of RNF6 up‐regulated the protein level of SHP‐1 in traumatic tissues and thus suppressed the activation of STAT3 by JAK. To summarize, we reported that inhibition of RNF6 alleviated TBI by suppressing the STAT3 signaling in TBI rats.

## 2 METHODS

### 2.1 Animals

Sixty‐four adult male (aged approximately 18 weeks) rats weighing between 260 and 320 g were purchased from Beijing Charles River Co., Ltd. All rats used in this research were cultured in the virus/antigen‐free system with permanent humidity and constant temperature and free access to pathogen‐free food and water. Animal studies were approved by the ethics commitment of Cangzhou Central Hospital.

As mentioned in previous research, cortical impact injury (CCI) was used to establish the TBI rat model. Forty rats were used for CCI treatment, and four rats died during operation. 2% isoflurane and oxygen were used to anesthetize the rats. The skin and periosteum in the midline of the rat brain were then sliced until the parietal bone was exposed. A surgical drill was applied to make a small hole behind the coronary suture, and a hydraulic device was connected to the brain tissue to make continuous pressure. The same operation was performed in the sham‐operated group of rats without the pressure.

The siRNA targeting RNF6 (AGAGAACGCAGAGGCACCGAUUAUA) was purchased from Guangdong Ribo Co., Ltd. siRNA targeting RNF6 (100 pmol in 2 μl) was injected into the basal ganglia 24 hr after the surgery, and 5% sodium bicarbonate solution was used as a negative control.

### 2.2 Brain water content assay

The brain water content was detected to evaluate the brain edema at day 5 after TBI. The brain water weight of rats was measured under former instruction. TBI and Sham rats were sacrificed, and their brains were removed from the skull immediately. The collected cerebral tissues were then cut into sections with a side length of 4 mm. And isometric cerebellum tissues were also collected as the internal control. An electronic analytical balance was used to weigh the tissues immediately, and the weight was recorded as wet weight (WW). Then, all tissue sections were placed in a dryer at 100°C for 24 hr. The tissues were then weighed by the same analytical balance, and the weight was recorded as dry weight (DW). Thus, the brain water content was calculated as (WW − DW)/WW × 100%.

### 2.3 Evans blue staining assay

Evans blue permeability was detected to evaluate the brain edema at day 5 after TBI. Rats were anesthetized with 10% chloral hydrate (0.3 ml/100 g). Evans blue (EB) (2%, 2 ml/kg) was injected from the tail vein 0.5 hr before the perfusion. The EB leakage through the blood–brain barrier (BBB) was used to assess the permeability of the BBB. Rats were perfused through the heart with 0.9% NaCl until the lavage fluid from the right atrium was clear. The brain tissue of the rat was removed. Each sample was weighed and then homogenized in 0.75 ml of PBS and 0.25 ml of 100% TCA solution. The sample was cooled at 4°C overnight and then centrifuged at 1,000 *g* for 30 min at 4°C. Subsequently, the EB in the 100 μl supernatant of each sample was measured at 620 nm using a 96‐well plate reader.

### 2.4 Modified neurological severity scores (mNSS)

The mNSS test was performed to score the neurobehavior of TBI rats at day 5 after the TBI surgery (Chen et al., 2001). The motor, sensory, reflex, and balance abilities of each rat were estimated and a higher score represented a worse neurofunction. Neurological function was graded on a scale of 0 to 18 (normal score, 0; maximal deficit score, 18). The tests included: motor tests (six points); sensory tests (two points); beam balance tests (six points); and reflexes absent and abnormal movements (four points). One point was awarded for inability to perform the tasks or for lack of a tested reflex. All scores used in this research were recorded and analyzed by investigators who were blind to the grouping of the rats.

### 2.5 Western blot assay

Five days after TBI treatment, the rat brain tissues were harvested and prepared for Western blot to detect protein expression. Radioimmunoprecipitation assay buffer was used to digest the cerebral tissue from the plates, and 2 × Loading buffer was used to extract proteins from each sample. The Bicinchoninic Acid Protein Assay Kit (Thermo Fisher Labsystems) was then used to quantify the concentrations of proteins for all samples. A 12% sodium dodecyl sulfate–polyacrylamide gel electrophoresis (SDS‐PAGE) was used to separate different proteins in these samples with a voltage of 140 V. And the proteins in the SDS‐PAGE were then transferred onto a polyvinylidene difluoride membrane (Thermo Fisher Labsystems) with an electric current of 300 mA. The membrane was coincubated with anti‐RNF6, anti‐STAT3, anti‐p‐STAT3, anti‐SHP‐1, and anti‐GAPDH (1:1,000, Abcam, Shanghai, China) at 37℃ for 1 hr and washed by PBS buffer for three times. Then, goat anti‑rabbit IgG (1:5,000, Abcam) was incubated with the membrane at 37°C for 1 hr and an ECL Western Blotting Substrate kit (Abcam, Shanghai, China) was used for chemiluminescence imaging.

### 2.6 QRT‐PCR assay

Five days after TBI treatment, the rat brain tissues were harvested and prepared for qRT‐PCR. The total RNA of rat cerebral tissue was extracted using a TRIzol Kit (Thermo Fisher Labsystems). Then, a SuperScript IV Kit (Thermo Fisher Labsystems) was used to reversely transcribe the mRNA of each sample to cDNA for further experiment. RNA levels of IL‐6, IL‐1β, TNF‐α, and IL‐10 were examined by real‐time PCR mix, and a S1000 PCR Thermal cycler was used to detect the system. GAPDH served as the loading control.

The primers were as follows:

IL‐1β: CTGTGACTCATGGGATGATGATG; CGGAGCCTGTAGTGCAGTTG

IL‐6: TCTATACCACTTCACAAGTCGGA; GAATTGCCATTGCACAACTCTTT

TNF‐α: CCTGTAGCCCACGTCGTAG; GGGAGTAGACAAGGTACAACCC

IL‐10: CAGTCAGCCAGACCCACAT; GGCAACCCAAGTAACCCT

RNF6: GCACCATTACAGTTCCTCTT; CACTCACATCTGGCATTTC

GAPDH: AATGGATTTGGACGCATTGGT; TTTGCACTGGTACGTGTTGAT

### 2.7 Statistical analysis

All experiments in this research were repeated independently at least three times for the accuracy of the data, which were represented as mean ± standard deviation (*SD*). The GraphPad Prism v7.0 software (GraphPad Software, Inc.) was used to analyze the raw data and conduct curves and histogram. *p* < .05 was considered as significant difference in this research (*p < .05, **p < .01, ***p < .001). Because a few data in the experiments were not appropriate for normal distribution (Kolmogorov–Smirnov *p* < .05), the differences in means among multiple groups were analyzed using one‐way ANOVA (nonparametric analyses, Kruskal–Wallis test) followed by Dunn's multiple comparisons test.

## 3 RESULTS

### 3.1 RNF6 was up‐regulated in the cerebral tissues in TBI rats

**Figure 1:**
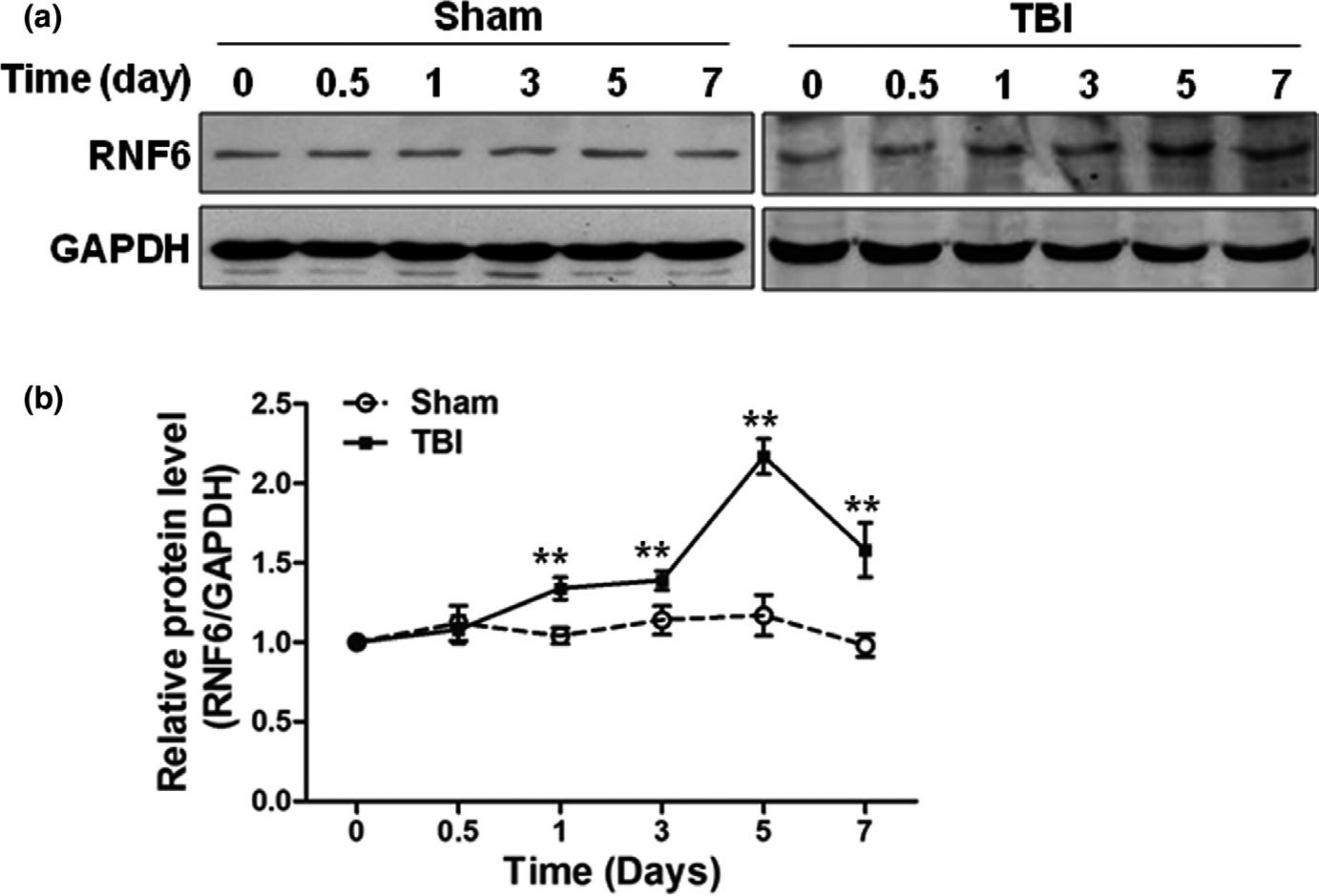
RNF6 is up‐regulated after TBI in rats. Western blot was used to detect RNF6 expression in sham‐operated group (sham) or TBI group at days 0, 0.5, 1, 3, 5, and 7 after operation (a). The optical density was also analyzed (b). Three rats were examined at each time point. TBI increased RNF6 expression in rat brain tissues. One‐way ANOVA (nonparametric analyses, Kruskal–Wallis test: d: *p* < .0001, Kruskal–Wallis statistic: 15.17.) followed by Dunn's multiple comparisons test was used. d: day 1: *p* = .0241, day 3: *p* = .0183, day 5: *p* = .0074, day 7: *p* = .01412 compared to day 0

To investigate whether TBI could induce the up‐regulation of RNF6 in the cerebral tissues, we first established the TBI rat model by CCI. The alteration in RNF6 protein level postinjury was estimated by Western blot assay. The protein levels of RNF6 at days 0, 0.5, 1, 3, 5, and 7 postsurgery of rats in the Sham group were shown in Figure 1a,b, and there was no significant difference. However, in the TBI group, the protein levels of RNF6 significantly increased at days 1, 3, and 5 post‐CCI (Figure 1a,b), indicating that TBI was a factor to up‐regulate the expression of RNF6 in traumatic cerebral tissues.

### 3.2 Inhibiting RNF6 alleviated the brain edema and improved the neurological outcome in TBI rats

**Figure 2:**
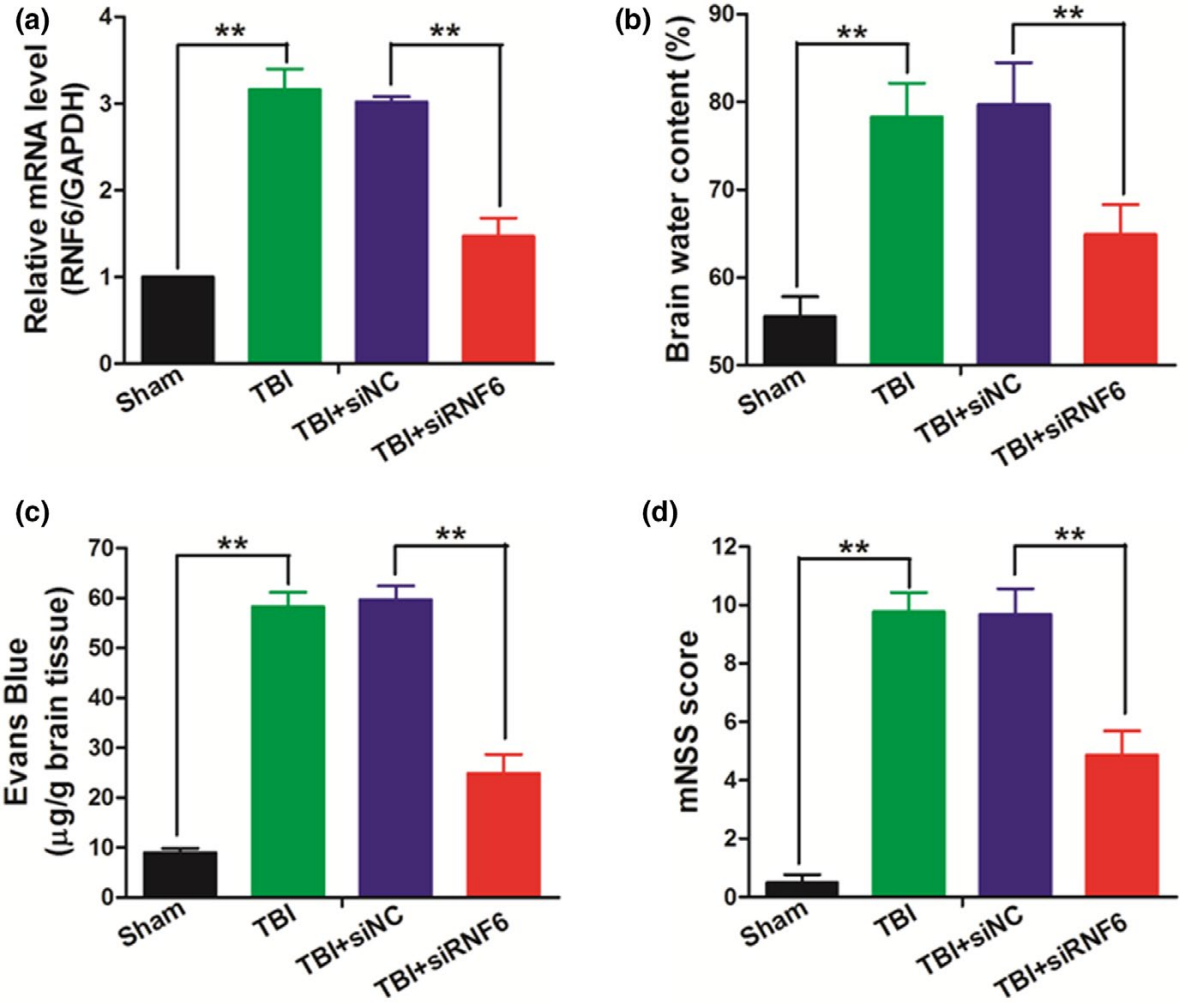
Inhibiting RNF6 alleviates the brain edema and improves the neurological outcome after TBI in rats. (a) QRT‐PCR was used to detect the knockdown efficiency of RNF6 at day 5 after TBI (*n* = 6/group). The brain water content (b) and Evans blue permeability (c) were detected to evaluate the brain edema at day 5 after TBI (*n* = 6/group). (d) The neurological outcome was evaluated by mNSS at day 5 after TBI (*n* = 6/group). One‐way ANOVA (nonparametric analyses, Kruskal–Wallis test: a: *p* = .0042, Kruskal–Wallis statistic: 17.43, b: *p* = .0002, Kruskal–Wallis statistic: 19.47, c: *p* = .0002, Kruskal–Wallis statistic: 19.66, d: *p* = .0015, Kruskal–Wallis statistic: 18.70.) followed by Dunn's multiple comparisons test was used. a: *p* = .0052, b: *p* = .0084, c: *p* = .0061, d: *p* = .0078 between TBI + siNC and TBI + siRNF6

To investigate the regulatory function of RNF6 in the progression of TBI, siRNA targeting RNF6 was used to knock down RNF6 in the brain tissues of rats. As shown in Figure 2a, TBI induced the up‐regulation of RNF6 which was inhibited by RNF6 siRNA. And RNF6 inhibition induced significantly decreased brain water content (Figure 2b) in the traumatic brain tissues and improved the integrity of BBB postinjury (Figure 2c). The neurofunction of rats post‐TBI was measured by mNSS test, and as shown in Figure 2d, rats treated with siRNA targeting RNF6 exhibited better neural behaviors than the siNC group, indicating that inhibition of RNF6 promoted the neuro‐recovery of TBI rats.

### 3.3 Inhibiting RNF6 suppressed the inflammation in TBI rats

**Figure 3:**
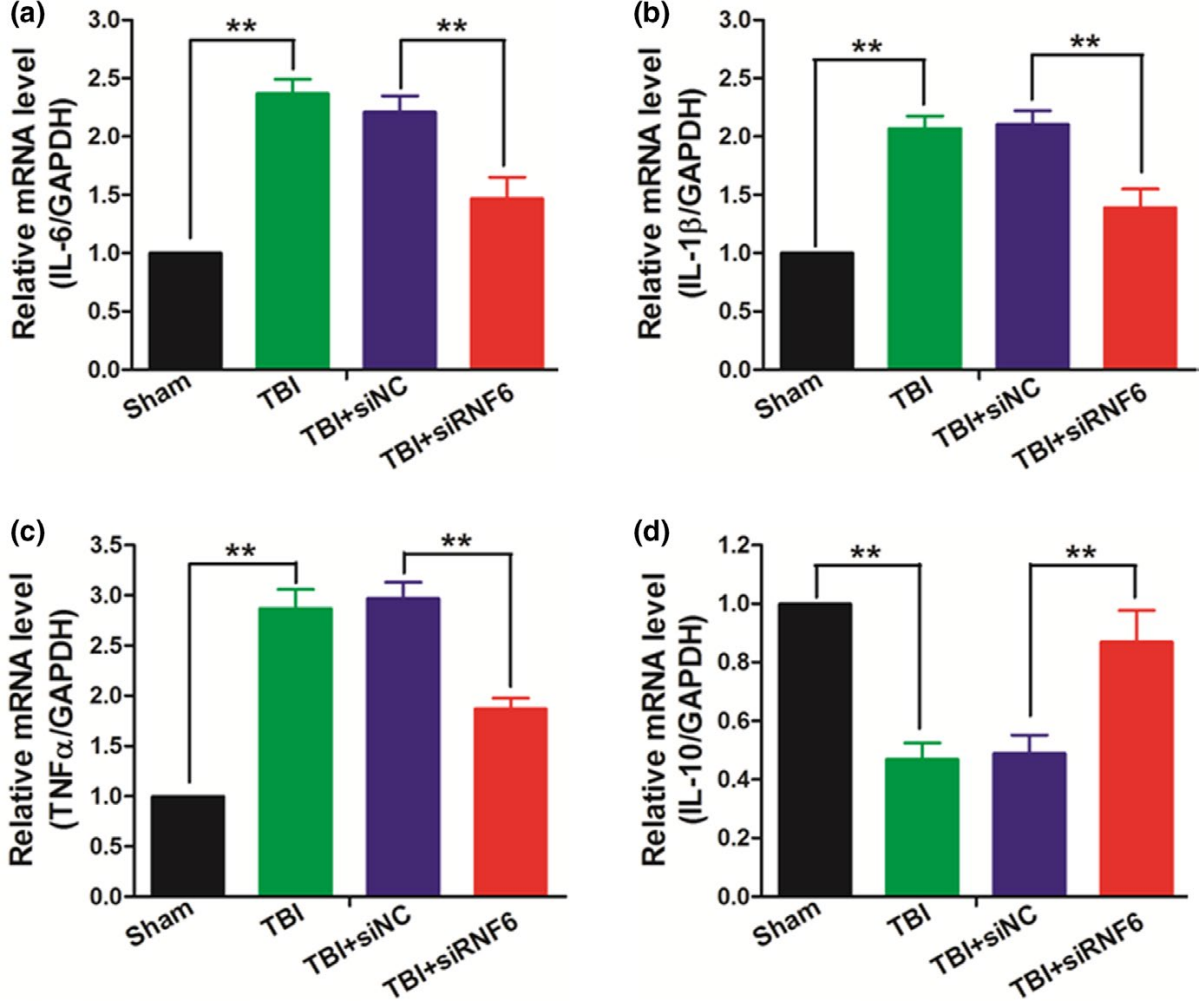
Inhibiting RNF6 suppresses inflammation after TBI in rats. QRT‐PCR was performed to detect the expression of IL‐6 (a), IL‐1β (b), TNFα (c) and IL‐10 (d). One‐way ANOVA (nonparametric analyses, Kruskal–Wallis test: a: *p* < .0001, Kruskal–Wallis statistic: 21.25, b: *p* = .0005, Kruskal–Wallis statistic: 20.00, c: *p* = .0001, Kruskal–Wallis statistic: 20.28, d: *p* = .0002, Kruskal–Wallis statistic: 19.38.) followed by Dunn's multiple comparisons test was used. a: *p* = .0086, b: *p* = .0075, c: *p* = .0063, d: *p* = .0036 between TBI + siNC and TBI + siRNF6

Given the crucial role that inflammation played in the progression of TBI, the serum cytokine expression levels were also estimated by qRT‐PCR assays in this research. The RNA levels of proinflammatory factors including IL‐6 (Figure 3a), IL‐1β (Figure 3b), and TNF‐α (Figure 3c) all increased postinjury and decreased after the treatment of siRNA targeting RNF6, suggesting that inhibition of RNF6 suppressed the cerebral inflammation. Consistently, the anti‐inflammation factors like IL‐10 were also up‐regulated with the treatment of siRNA (Figure 3d).

### 3.4 Inhibiting RNF6 up‐regulated the SHP‐1 expression in TBI rats

**Figure 4:**
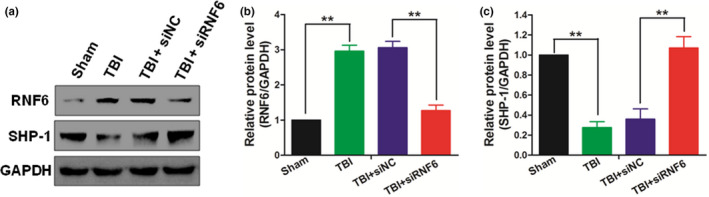
Inhibiting RNF6 upregulates SHP‐1 expression after *TBI in rats*. (a) After TBI treatment for 5 days, the expression levels of RNF6 and SHP‐1 were detected by Western blot. The expression levels of RNF6 (b) and SHP‐1 (c) were analyzed by optical density. One‐way ANOVA (nonparametric analyses, Kruskal–Wallis test: b: *p* = .0003, Kruskal–Wallis statistic: 20.03, c: *p* = .0002, Kruskal–Wallis statistic: 20.24.) followed by Dunn's multiple comparisons test was used. b: *p* = .0055, c: *p* = .0016 between TBI + siNC and TBI + siRNF6

Since we have demonstrated the proinflammatory function of RNF6 in the progression of TBI, it was then necessary to explore the detailed mechanism underlying this process. We found that the up‐regulation of RNF6 induced by TBI decreased the protein level of SHP‐1 (Figure 4a). TBI induced the up‐regulation in the protein level of RNF6 which could be reduced by siRNA targeting RNF6 (Figure 4b). The up‐regulated RNF6 mediated the degradation of SHP‐1 during the progression of TBI (Figure 4c). Given that SHP‐1 participated in the regulation of STAT3 signaling and impacted the regional inflammation, it was possible that RNF6 promoted the inflammatory reaction in TBI rats through SHP‐1.

### 3.5 Inhibiting RNF6 suppressed STAT3 activation post‐TBI

**Figure 5:**
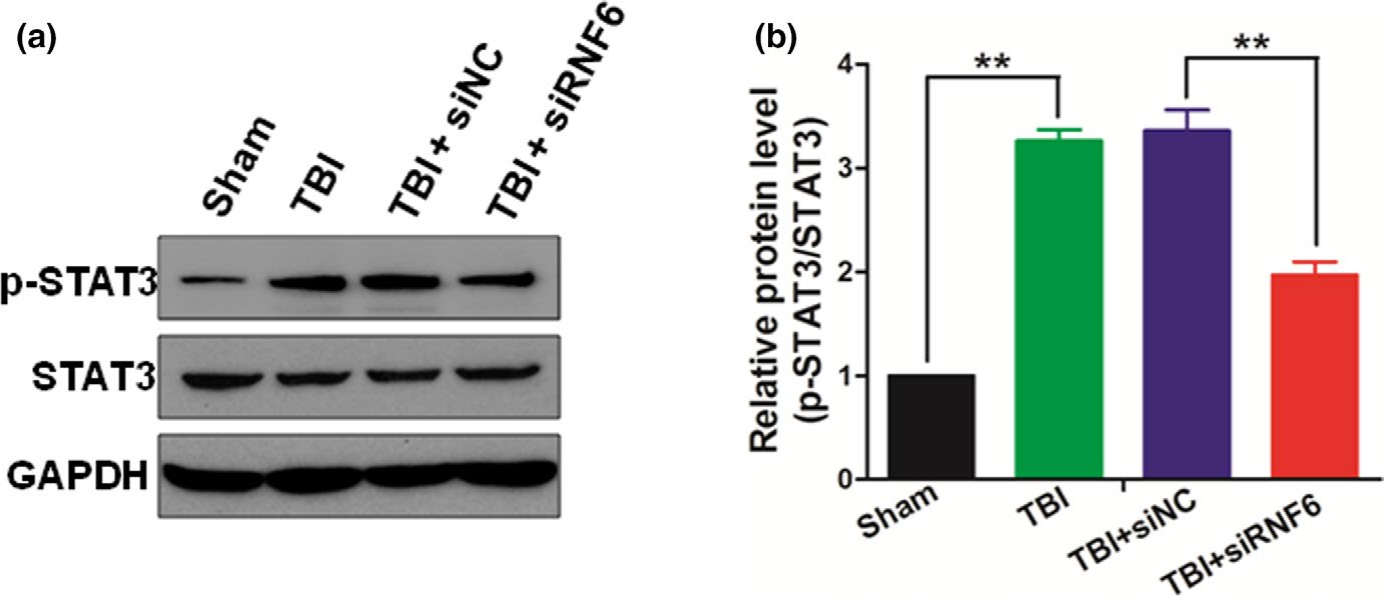
Inhibiting RNF6 suppresses STAT3 activation after TBI in rats. (a) After TBI treatment for 5 days, the expression levels of p‐STAT3 and STAT3 were detected by Western blot. (b) The relative expression levels of p‐STAT3 were analyzed by optical density. One‐way ANOVA (nonparametric analyses, Kruskal–Wallis test: b: *p* = .0003, Kruskal–Wallis statistic: 18.74.) followed by Dunn's multiple comparisons test was used. b: *p* = .0043 between TBI + siNC and TBI + siRNF6

The RNF6 in the brain tissues of TBI rats promoted the degradation of SHP‐1. Thus, Western blot assay was performed to investigate whether the up‐regulated RNF6 in TBI rats could influence the JAK/STAT3 signaling. As shown in Figure 5a,b, TBI increased the phosphorylation level of STAT3 which could be decreased by siRNA against RNF6, while the total STAT3 level remained unchangeable during this process. This result thus suggested that inhibition of RNF6 alleviated the inflammation of TBI rats through suppressing the STAT3 signaling pathway.

## 4 DISCUSSION

TBI remains a significant clinical challenge in spite of several breakthroughs of trauma management strategies in recent years (Donnelly, Young, & Brady, 2017). The incidence of TBI is up to 2% ‐ 3% in the industrialized countries in Europe, and it has ranked as one of the leading causes of morbidity and mortality of adolescent and adults under 40 years of age all over the world (Bomyea, Lang, & Schnurr, 2017). It is estimated that about 52,000 people die from TBI each year in the United States, and about 530,000 patients with TBI end up with permanent disability (Miles et al., 2017). In developed countries, the average age of TBI patients is increasing. And falls have surpassed traffic accidents to become the main cause of TBI (Wang et al., 2018). In general, there are two clinical stages of brain trauma: primary injury and secondary injury. Primary injury is induced by direct impact of external force or deformation of tissue caused by acceleration, deceleration, or rotation, resulting in dysfunction of the brain tissue and cells (Noggle & Pierson, 2010). Although we can use some protective or preventive measures to avoid brain trauma, this transient injury is irreversible once TBI occurs. The lasting and long‐term secondary injury is composed of brain edema, cerebral infraction, intracranial hypertension, neural inflammation, necrosis, hydrocephalus, and BBB dysfunction, which makes the management of TBI more challenging and complex than the primary structure destruction caused by TBI itself (Oberholzer & Muri, 2019). These damages can lead to irreversible death of nerve cells. Therefore, alleviating secondary injury is the key to treat TBI (Noggle & Pierson, 2010).

Neuroinflammation is an important part of TBI secondary injury. Glial cells and neurons in the inflammatory state release endogenous substances and induce the activation of the innate immune system, which plays a key role in the recovery of TBI. However, the dysregulation of immune response can also lead to secondary damage to the central nervous system (Gardner et al., 2018). Cytokines are released from neutrophils, microglia, immune, and glial cells in the injured area, including anti‐inflammatory factors such as IL‐10, transforming growth factor‐β, neurotrophic factors, IL‐4, IL‐13, prostaglandins, and proinflammatory substances such as IL‐10, CXCL1, IL‐1, IL‐6, and TNF‐α. Accumulation of these factors leads to the overreaction of neutrophils, which causes the destruction of BBB integrity and excessive neural apoptosis (Anderson, Parmenter, & Mok, 2002). Thus, inhibiting the inflammatory reaction during the progression of TBI is essential for its treatment. In this research, we reported that RNF6 could promote the inflammation in the traumatic brain areas in TBI rats. We demonstrated that the up‐regulated RNF6 mediated the degradation of SHP‐1, and thus activated the STAT3 signaling pathway in TBI rats. Therefore, inhibition of RNF6 could be a potential therapy for TBI by inhibiting the cerebral inflammation mediated by the STAT3 signaling pathway.

The JAK/STAT3 signaling pathway plays a significant role in cell proliferation, differentiation, apoptosis, and inflammation through various factors (Shi et al., 2016). The activation of JAK/STAT3 pathway promotes the occurrence and development of various diseases, including inflammatory diseases, lymphomas, leukemias, and solid tumors (Bhat, Goel, Shukla, & Hanif, 2016). Targeted therapy for the JAK/STAT3 signaling pathway has been a hot research spot currently. Previous research has demonstrated the correlation between STAT3 and neuroinflammation in TBI (Shi et al., 2016). JAKs usually bind to intracellular cytokine membrane receptors and are phosphorylated to be activated. The activated JAKs catalyze the phosphorylation of STAT3 family members to form homodimers, which are transferred to the nucleus and bind to specific DNA sequences to induce selective transcription activation (Dominguez, Mauborgne, Mallet, Desclaux, & Pohl, 2010). The expression levels of JAK and STAT3 are significantly increased under the TBI state, especially in the brain astrocytes and microglia (Zong et al., 2014). These studies indicate that the JAK/STAT3 pathway plays a key role in the inflammatory response of cerebral trauma. Brain injury is usually accompanied by oxidative stress, which will also increase cerebral ischemia and neurodegeneration (Jiang & Tang, 2020; Zeng, Wang, Dong, Jiang, & Tu, 2014). Previous studies in the mouse TBI model have found that STAT3 could also be activated by the stimulation of reactive oxygen species (Saleem et al., 2020). It has been reported that, in the brain astrocytes, hydrogen peroxide intervention can increase the phosphorylation levels of STAT1 and STAT3 (Yu et al., 2020), both of which have also been found to be regulated by JAK2 (Zheng et al., 2019). Activated STAT3 translocates into the nucleus and activates the γ‐active site of DNA, thus inducing the apoptosis of astrocytes (Zhou et al., 2019).

In this research, we reported that RNF6 could induce the activation of STAT3 in TBI rats, which worsened the inflammatory reaction and destroyed the integrity of BBB. By inhibiting the expression of STAT3 in TBI rats, the STAT3 signaling pathway was repressed and the symptoms of TBI were alleviated. Thus, we believe that RNF6 inhibition could play an important role in the clinical management of TBI patients.

Intracellular protein ubiquitination degradation is one of the important posttranscriptional modifications of proteins. The ubiquitinated substrate and its subsequent degradation process exist in the plasma membrane system of the entire cell, including cell membrane, the endoplasmic reticulum, and the nuclear membrane (Li, Yu, Sun, & Li, 2015). The changes in cellular ubiquitination and de‐ubiquitination are closely related to the occurrence of tumors and other diseases (Choi, Park, Lee, Han, & Choi, 2013). The proteins modified by polyubiquitination are recognized by the proteasome and then degraded. Three key enzymes jointly mediate this polyubiquitination process, including ubiquitin‐activating enzyme E1, ubiquitin binding enzyme E2, and ubiquitin ligase E3. Compared with E1 and E2, ubiquitin ligase E3 can specifically participate in substrate recognition (David, Nair, & Pillai, 2013). Thus, the specificity of the ubiquitin–proteasome system to degrade target proteins is determined by ubiquitin ligase E3, which attracts great research attention. RNF6 is a type of E3 ligase, and previous research has demonstrated that RNF6 can specifically recognize SHP‐1 and mediate its degradation (Huang et al., 2018). And the effect of SHP‐1 on the dephosphorylation of the 705‐tyrosine residue of STAT3 plays a crucial role in keeping the STAT3 activity low (Beldi‐Ferchiou et al., 2017). Thus, RNF6 could regulate the STAT3 signaling by promoting the degradation of SHP‐1. In this research, we also demonstrated that RNF6 mediated the protein degradation of SHP‐1 and thus activated the STAT3 signaling pathway.

## 5 CONCLUSION

In conclusion, we reported that inhibition of RNF6 alleviated TBI by suppressing the STAT3 signaling in TBI rats. We first demonstrated that RNF6 was up‐regulated in traumatic cerebral tissues and investigated the regulatory role of RNF6 in the progression of TBI. Inhibiting RNF6 not only reduced the incidence of brain edema caused by TBI but also protected the integrity of BBB and the function of central nerves system. Inhibition of RNF6 alleviated the inflammation in TBI rats by up‐regulating the protein level of SHP‐1 and thus inhibiting the activation of the JAK/STAT3 signaling. We believe our study provides a potentially new strategy for the management of TBI patients in clinic.

## CONFLICTS OF INTEREST

The authors declare that they have no conflict of interest.

## **AUTHOR** **CONTRIBUTION**

B.L, G.Z, S.C, and G.D conducted the experiments. B.L and G.Z analyzed the data and wrote the manuscript. B.L conceived the study.

### Peer Review

The peer review history for this article is available at https://publons.com/publon/10.1002/brb3.1847.

## Data Availability

Data could be obtained upon reasonable request to the corresponding author.
